# Nonagenarians with major neurocognitive disorder hospitalized in a high-complexity hospital

**DOI:** 10.1590/1980-5764-DN-2025-0447

**Published:** 2026-10-02

**Authors:** Elly Morros-González, Santiago Pérez, Daniela Arias-Blanco, María Fernanda Duenas-Orejuela

**Affiliations:** 1 Hospital Universitario Mayor-Méderi, Instituto Méderi del Envejecimiento y Longevidad, Bogotá, Colombia.; 2 Universidad del Rosario, Escuela de Medicina y Ciencias de la Salud, Bogotá, Colombia.

**Keywords:** Dementia, Neurocognitive Disorders, Nonagenarians, Hospitalization, Colombia, Demencia, Trastornos Neurocognitivos, Nonagenarios, Hospitalización, Colombia

## Abstract

The prevalence of neurocognitive disorder increases with age; nearly one in two nonagenarians may be affected, representing a clinical challenge during hospitalization because of their admission condition, comorbidities, decision-making needs, and management.

**Objective:**

This study aimed to describe the clinical characteristics of individuals aged 90 years old or older with a history of major neurocognitive disorder admitted due to an acute medical condition.

**Methods:**

A descriptive cross-sectional study was conducted using institutional data from the Geriatrics Department of a high-complexity hospital in Bogotá, Colombia. Information on the history and type of neurocognitive disorder was obtained through self-report or proxy report, and disease severity was assessed using information recorded during the comprehensive geriatric assessment. A descriptive analysis was performed to examine clinical characteristics, baseline functional status, and in-hospital outcomes.

**Results:**

Among the 178 people, the majority were women (71.34%), with a mean age of 93±3 years, and had polypharmacy. Upon admission, 55.61% had delirium, and among the 68 patients who underwent nutritional assessment, 75.00% had moderate or severe malnutrition. The most frequent diagnoses were urinary tract infection and pneumonia. Alzheimer disease (62.5%) and multiple etiologies (Alzheimer disease and vascular) (6.2%) were the most frequent reported etiologies. The median length of stay was 7 days, 4.49% experienced in-hospital complications such as healthcare-associated pneumonia or bacteremia, 45.51% continued management through a home care plan, and 34.83% completed the planned hospital care.

**Conclusion:**

Nonagenarians with major neurocognitive disorder have multiple healthcare needs during hospitalization, requiring multidimensional and interdisciplinary assessment and intervention.

## RESUMEN

La prevalencia del trastorno neurocognitivo aumenta con la edad; casi una de cada dos personas nonagenarias puede verse afectada, lo que representa un desafío clínico durante la hospitalización debido a la condición de ingreso, las comorbilidades, las necesidades de toma de decisiones y el manejo clínico.

**Objetivo:**

Describir las características clínicas de personas de 90 años o más con antecedentes de trastorno neurocognitivo mayor (TNM) ingresadas por una condición médica aguda.

**Métodos:**

Se realizó un estudio descriptivo de corte transversal utilizando datos institucionales del servicio de geriatría de un hospital de alta complejidad en Bogotá, Colombia. La información sobre el antecedente y el tipo de trastorno neurocognitivo se obtuvo mediante autoinforme o información proporcionada por un informante, y la gravedad de la enfermedad se evaluó a partir de la información registrada durante la valoración geriátrica integral. Se realizó un análisis descriptivo para examinar las características clínicas, el estado funcional basal y los desenlaces intrahospitalarios.

**Resultados:**

Entre los 178 pacientes, la mayoría eran mujeres (71,34%), con una edad media de 93±3 años y polifarmacia. Al ingreso, el 55,61% presentaba delirium y, entre los 68 pacientes sometidos a evaluación nutricional, el 75,00% presentaba desnutrición moderada o grave. Los diagnósticos más frecuentes fueron infección del tracto urinario y neumonía. La enfermedad de Alzheimer (62,5%) y las etiologías múltiples (enfermedad de Alzheimer y enfermedad vascular) (6,2%) fueron las etiologías notificadas con mayor frecuencia. La mediana de la estancia hospitalaria fue de 7 días; el 4,49% presentó complicaciones intrahospitalarias, como neumonía asociada a la atención en salud o bacteriemia; el 45,51% continuó el manejo mediante un plan de atención domiciliaria y el 34,83% completó la atención hospitalaria planificada.

**Conclusión:**

Los nonagenarios con trastorno neurocognitivo mayor presentan múltiples necesidades de atención durante la hospitalización, lo que requiere una evaluación e intervención multidimensional e interdisciplinaria.

## INTRODUCTION

The global population aged 65 years old and older has grown rapidly, from 761 million in 2022, and is expected to reach 1.6 billion by 2050 (World Health Organization – WHO)^1^. In Colombia, this group represents 9.1% of the population, compared to 4.9% in 1964 (National Administrative Department of Statistics/*Departamento Administrativo Nacional de Estadística* – DANE)^2^. This increase reflects greater life expectancy and a rise in chronic diseases such as major neurocognitive disorder (MND).

According to the WHO, an estimated 55 million people worldwide live with some form of neurocognitive disorder, with a global prevalence ranging between 5 and 7%^3^, although this figure varies considerably by region. For instance, in Latin America, the prevalence reaches 8.5% among individuals over 60 years of age, and in some Colombian cities, such as Neiva, it can be as high as 23%^4^. It is important to note that these predominantly reflect adults aged 60 years old and older, as population-specific prevalence estimates for nonagenarians remain scarce in the literature. In a high-complexity hospital in Bogotá, the prevalence of cognitive impairment among nonagenarians was 17.10%^5^.

The high prevalence of this condition has led to an increasing demand for medical care. In response, various strategies have been proposed to strengthen healthcare networks within health systems, aiming to ensure comprehensive care for these patients across different levels of care. One notable example is home-based care, which has shown significant benefits in improving the quality of life of this population^6^. However, inpatient care for individuals with MND poses a major challenge for healthcare personnel, as these patients require complex and multidimensional management due to multiple comorbidities^7,8^. Furthermore, identifying the condition leading to hospitalization is often difficult, since admissions are frequently associated with delirium^9^. During hospitalization, patients with MND are also at high risk of adverse events such as falls^10^, hospital-acquired infections, and pressure injuries^11^, further complicating their clinical management and demanding specialized care to minimize complications.

Among nonagenarians (≥90 years), these challenges are further intensified by their distinctive clinical profile. This population exhibits a disproportionately high burden of multimorbidity, advanced frailty, severe functional dependence, sarcopenia, and sensory impairment, contributing to marked clinical vulnerability. In individuals with MND, cognitive decline is often more advanced, with a higher frequency of mixed etiologies and increased susceptibility to delirium, malnutrition, and infectious complications during hospitalization^5,12^.

Importantly, both the prevalence and clinical expression of MND appear to differ substantially in this age group compared with younger older adults. In very advanced age, the interaction between frailty and cognitive decline becomes particularly pronounced and is likely bidirectional, shaping disease trajectories, resilience to acute stressors, and in-hospital outcomes. These complexities also influence decision-making processes, which frequently require surrogate involvement and greater emphasis on goal-concordant or palliative approaches. Collectively, these factors underscore the need for explicit consideration of nonagenarians as a distinct clinical subgroup in the inpatient setting^13,14^.

Despite the increasing prevalence of MND, direct evidence specifically examining the clinical and psychosocial characteristics of hospitalized nonagenarians with MND remains scarce, particularly in Latin American settings^5,13^. This knowledge gap limits a comprehensive understanding of their needs in high-complexity healthcare environments. Therefore, this study aimed to describe the clinical characteristics, baseline functional status, and psychosocial aspects of nonagenarian patients with a history of MND hospitalized for clinical causes at a single high-complexity hospital.

## METHODS

We conducted a descriptive cross-sectional observational study using data from the AGING registry, an active cohort-type registry conducted at a single high-complexity hospital in Bogotá, Colombia, that provides care to both public and private patients and was initiated in November 2022. The registry prospectively captures clinical and functional data from adults aged 90 years old or older hospitalized under the care of the geriatrics service at this institution for research and clinical follow-up purposes, supported by the Centro de Investigaciones Méderi (CIMED). All variables are derived from the comprehensive geriatric assessment (CGA) performed at hospital admission by geriatricians, reflecting the patient’s baseline status during the 15 days prior to the onset of the condition leading to hospitalization, and are recorded by a trained research assistant, with periodic audits conducted by both CIMED and the research group.

From the first 1,000 patients enrolled, we included adults aged 90 years old or older with a history of neurocognitive disorder, identified through self-report or proxy report by a family member at hospital admission who could provide reliable information, most frequently the primary caregiver. This was a convenience sample through January 2024. Severity of neurocognitive disorder was assessed using information from the CGA and, when available, documented Global Deterioration Scale (GDS) or Clinical Dementia Rating (CDR) scores during hospitalization. Patients referred from other hospitals and those whose hospitalization ended prematurely for administrative reasons were excluded.

### Variables of interest

Sociodemographic and clinical variables were documented. To assess baseline status, the Barthel Index^15^ was used to evaluate basic activities of daily living, defining the following categories: independent (score ≥90), mild dependency (60–85), moderate dependency (40–55), severe dependency (20–35), and total dependency (0–15). The Lawton and Brody scale^16^ was applied to assess instrumental activities of daily living, defining autonomy as a score of 8, mild dependence as 6–7, moderate dependence as 4–5, severe dependence as 2–3, and total dependence as 0–1.

Frailty was assessed using the Clinical Frailty Scale (CFS)^17,18^, a tool that describes different degrees of individual vulnerability, with scores ranging from robustness (1 point) to terminal illness (9 points). Frailty was defined as a CFS score ≥5. GDS scores of 2–3 were interpreted as mild MND, 4–5 as moderate, and 6–7 as severe; CDR scores of 1 indicated mild stage, 2 moderate, and 3 severe.

Additionally, falls during the previous year were recorded. In the mental domain, the presence of delirium at admission was identified using the Confusional Assessment Method (CAM). For the purposes of this study, a total CAM score ≥ 3 was used as an operational threshold to define delirium, in addition to cases with a documented clinical diagnosis at admission^19,20^. Information regarding delirium subtype was not available.

In the nutritional domain, the Mini Nutritional Assessment–Short Form (MNA-SF)^21^ was used, where scores of 0–7 indicate malnutrition, 8–11 indicate risk of malnutrition, and 12 or higher indicate adequate nutritional status. Polypharmacy was defined as the use of five or more medications^22^.

Furthermore, referrals for social work, clinical nutrition (including whether nutritional supplementation was required), physical therapy evaluation, and the occurrence of adverse events during hospitalization were also recorded.

### Statistical analysis

Descriptive statistical methods were applied, using measures of central tendency and dispersion according to the type of variable. All analyses were performed using R Studio software, version 4.2.2. Additionally, the etiologies and severity of neurocognitive disorders were summarized and displayed in bar charts.

### Ethical considerations

This project was classified as minimal risk under Resolution 008430 of October 4, 1993, issued by the Ministry of Health of the Republic of Colombia. It also adhered to the ethical principles established in the Declaration of Helsinki and the Good Clinical Practice Guidelines of the International Conference on Harmonization. Approval was obtained from the Research Ethics Committee (*Comité de Ética en Investigación* – CEI) of Universidad del Rosario (DVO005 2865-CV1879), which granted a waiver of informed consent due to the nature of the study. The data were obtained from the AGING registry, which also had prior approval from the Human Research Ethics Committee (CEISH-2025002).

## RESULTS

From the information on the first 1,000 patients included in the AGING registry, 178 patients (17.8%) had a medical history of neurocognitive disorder. The majority were women (71.34%), with a mean age of 93±3 years. Regarding sociodemographic characteristics, most patients (98.31%) came from urban areas. In terms of education, 66.88% had completed primary education, and 66.29% lived with at least one child (Table 1).

**Table 1:** General and clinical characteristics of the patients.

Characteristics	n=178
Female sex, n (%)	127 (71.34)
Age, mean±SD	93±3 years
Urban residential area, n (%)	175 (98.31)
Educational level (n=154), n (%)	
Primary	103 (66.88)
Bachelor’s degree	28 (18.18)
Technical education	2 (1.29)
University education	5 (3.24)
No formal education	16 (10.38)
Lived with at least one child, n (%)	118 (66.29)
Comorbidities, median (IQR)	3 (2-4)
Hypertension, n (%)	132 (74.16)
COPD, n (%)	63 (35.39)
Hypothyroidism, n (%)	61 (34.26)
Number of medications used	
≥5 medications	119 (66.85)
Median (IQR)	6 (4–8)
Range	0–18
Hospitalization during the previous year, n (%)	51 (28.65)
Barthel Index (n=176)	
Median (IQR)	25 (10–40)
Independent, n (%)	3 (1.70)
Mild dependency, n (%)	21 (11.93)
Moderate dependency, n (%)	32 (18.18)
Severe dependency, n (%)	54 (30.68)
Total dependency, n (%)	66 (37.50)
Clinical Frailty Scale (n=156), n (%)	
Not frail (CFS 1-4)	5 (3.21)
Mildly frail (CFS 5)	15 (9.61)
Moderately frail (CFS 6)	40 (25.64)
Severely frail (CFS 7)	68 (43.58)
Very severely frail (CFS 8)	28 (17.94)
Terminally ill (CFS 9)	0
≥1 fall during the previous year, n (%)	63 (35.39)
Nutritional status (MNA-SF, n=124)	
Median (IQR)	8 (5–10)
Normal nutritional status (12–14)	55 (44.35)
At risk of malnutrition (8–11)	59 (47.58)
Malnourished (0–7)	10 (8.07)
Clinical nutrition assessment (n=68), n (%)	
Well nourished	4 (5.88)
Moderate malnutrition	21 (30.88)
Severe malnutrition	30 (44.12)
Obesity	4 (5.88)
Other	9 (13.24)
Received nutritional supplementation	49 (90.07)
**Delirium at admission, n (%)**	99 (55.61)

Abbreviations: n, sample size; %, percentage; SD, standard deviation; IQR, interquartile range; COPD, chronic obstructive pulmonary disease; CFS, Clinical Frailty Scale; MNA-SF, Mini Nutritional Assessment–Short Form.

The median number of comorbidities per patient was 3 (2–4), with hypertension being the most frequent (74.16%). The number of medications used per patient ranged from 0 to 18, with polypharmacy (≥5 medications) in 66.85% of patients, and 28.65% of patients had experienced at least one hospitalization in the year prior to admission (Table 1).

Additionally, 68 patients were evaluated by the clinical nutrition service, of whom 44.12% were diagnosed with severe malnutrition, 30.88% with moderate malnutrition, and 90.07% received a nutritional supplement prescription (Table 1).

After the comprehensive clinical assessment, poor or limited social support was suspected in 30 of the 178 patients. Following formal evaluation by the social work service, an adequate support network was identified. Furthermore, 55.05% of patients received at least one evaluation by the physical therapy service.

In relation to functional status, the median Barthel Index score was 25 points, and all evaluated patients presented total dependency according to the Lawton and Brody scale for instrumental activities of daily living. Frailty, as measured by the CFS, was present in 151 of 156 patients (96.79%), with severe frailty being the most prevalent category (43.58%) (Table 1).

The most frequently reported etiology of neurocognitive disorder among patients was Alzheimer disease, accounting for 62.50% of the total. This was followed by 23.9% of patients with an unknown etiology (Figure 1). Regarding disease severity, 55.1% had severe MND, 20.8% were at a moderate stage, and 23.0% had no stratified severity level (Figure 2). Finally, concerning the presence of delirium, more than half of the patients (55.61%) exhibited delirium at the time of hospital admission (Table 2).

**Figure 1:**
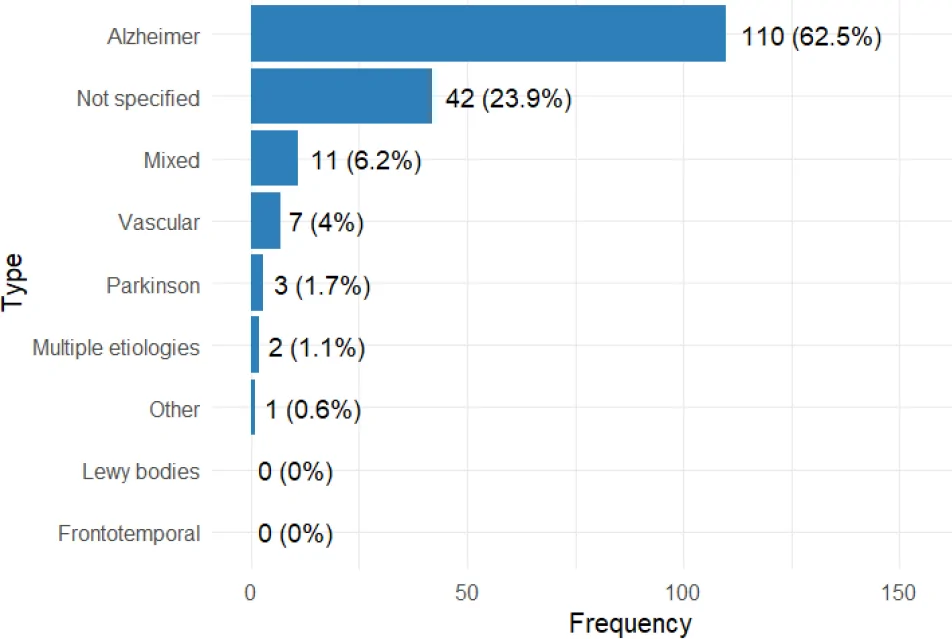
Clinical etiologies of major neurocognitive disorder, n=178.

**Figure 2:**
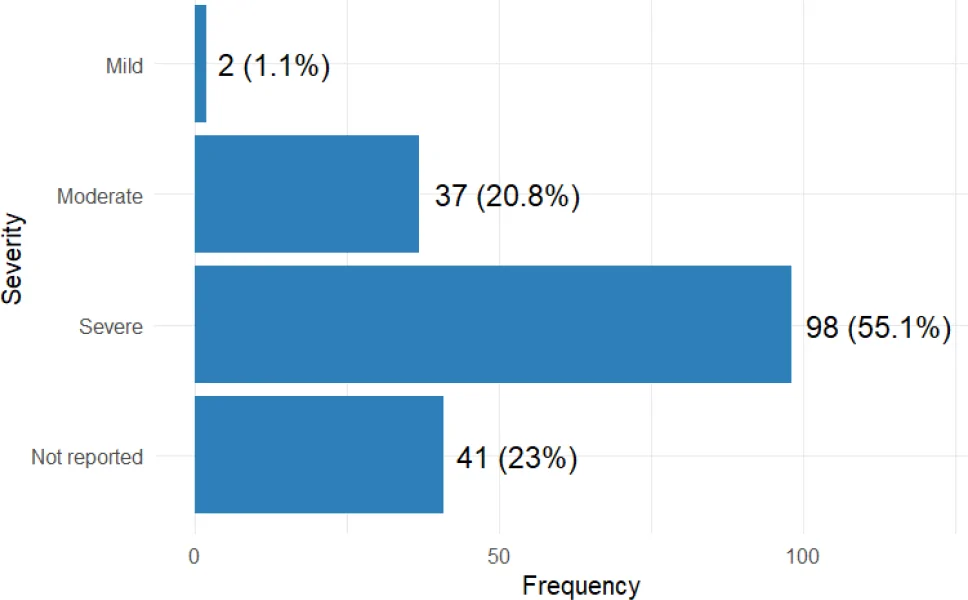
Clinical severity of major neurocognitive disorder, n=178. Severity was classified based on clinical impression and, when available, using the Global Deterioration Scale and Clinical Dementia Rating as follows: GDS scores of 2–3 were interpreted as mild major neurocognitive disorder, 4–5 as moderate, and 6–7 as severe; CDR scores of 1 indicated mild stage, 2 moderate, and 3 severe.

**Table 2:** Characteristics of the hospital stay.

Characteristics	n=178
Length of hospital stay, median (IQR)	7 (4–9) days
Incident in-hospital delirium (n=99), n (%)	17 (17.17)
Four most frequent discharge diagnoses	
Urinary tract infection, n (%)	31 (17.41)
Pneumonia, n (%)	29 (16.29)
Decompensated heart failure, n (%)	12 (6.74)
COPD, n (%)	10 (5.61)
In-hospital adverse events, n (%)	8 (4.49)
Pneumonia	5
Bacteremia	2
Phlebitis	1
Hospital disposition	
Completed planned hospital care, n (%)	62 (34.83)
Prolonged hospitalization, n (%)	11 (6.18)
Home care plan, n (%)	81 (45.51)
In-hospital death, n (%)	22 (12.35)
Other (institutional transfer and voluntary discharge), n (%)	2 (1.12)

Abbreviations: n, sample size; IQR, interquartile range; COPD, chronic obstructive pulmonary disease.

Regarding hospital stay, the median length of stay was 7 days, with an interquartile range (IQR) of 4–9 days, ranging from a minimum of 1 day to a maximum of 36 days. In terms of complications and clinical outcomes, incident in-hospital delirium occurred in 17.17% of patients. Infections were the most frequent discharge diagnosis, with urinary tract infections being the most common (17.41%), followed by pneumonia (16.29%). Among in-hospital adverse events, eight cases were recorded: five due to pneumonia, two due to bacteremia, and one due to phlebitis. At the end of hospitalization, 34.83% of patients completed their hospital stay satisfactorily, 45.50% were discharged to continue treatment at home, and 12.35% died during hospitalization (Table 2). Among these patients, 77.27% were female, 59.09% presented total dependence in basic activities of daily living, 68.18% had severe or very severe frailty, three-quarters had moderate to severe malnutrition after clinical nutrition assessment (75.00%), and 50.00% had a diagnosis of pneumonia at discharge.

## DISCUSSION

This study provides a detailed characterization of hospitalized nonagenarians with MND identified through self-report or proxy report by a family member and included in the AGING registry, who frequently exhibited severe dependence in basic activities of daily living, severe frailty, delirium at admission, and severe malnutrition.

The vast majority were women, which is consistent with findings reported in the literature, where a higher prevalence of MND has been observed among females^23^. However, the underlying cause of this phenomenon remains unclear, and several hypotheses have been proposed. One of them suggests that women’s greater longevity compared to men increases their risk of developing this condition due to longer exposure to aging-related processes^24^. Furthermore, 66.88% of the patients in this study had an educational level not exceeding primary school, which aligns with previous studies through the concept of cognitive reserve^25^ and reflects the cultural and historical characteristics of the period in which this population spent their childhood and adolescence^26^. These are explanatory hypotheses, and such relationships were not directly examined in the present analysis.

Regarding the clinical characteristics of the patients, polypharmacy was frequently observed (66.85%), with a wide range in the number of medications used and a maximum of 18 drugs recorded. According to the literature, polypharmacy is common among patients with MND^27^ and is associated with an increased risk of frailty^28^ and cognitive decline^29^. This highlights the importance of continuous medication review by geriatricians and other medical specialists, with special attention to the identification of potentially inappropriate prescriptions using validated tools^30,31,32,33^ and the adjustment of treatment plans according to each patient’s therapeutic goals.

In relation to the reported etiology of MND by family members, Alzheimer disease was the most commonly reported etiology, consistent with previous studies^34^. Specifically, it has been reported to account for 60–80% of all cases of MND^3^. However, identifying the underlying etiology is clinically relevant, as it may help anticipate the expected trajectory of cognitive decline, the likelihood of behavioral symptoms, and complementary management needs. In very old adults, mixed pathologies (particularly vascular contributions) are common and may be underrecognized when diagnoses rely on proxy reports. This consideration is particularly relevant in hospitalized nonagenarians, in whom vascular burden and multimorbidity frequently overlap with neurodegenerative processes, potentially influencing clinical presentation and outcomes.

Upon hospital admission, more than half of the patients with neurocognitive disorder presented with delirium, a condition frequently associated with this population. Delirium contributes to greater complexity in inpatient management, longer hospital stays, higher complication rates, and even increased mortality^9^.

In the nutritional assessment, moderate and severe malnutrition were highly prevalent in this study. This finding may be explained by the frequent association with dysphagia observed in the moderate and severe stages of the disease as part of its natural progression. Malnutrition has been shown not only to accelerate cognitive decline but also to worsen neuropsychiatric symptoms, such as agitation and depression^35^, and to increase mortality^36^. This highlights the importance of addressing and managing both malnutrition and swallowing disorders in this population, as they have a significant impact on quality of life and clinical outcomes.

Regarding frailty in individuals with neurocognitive disorders, although it may not be fully reversible, it remains modifiable. The relationship between frailty and cognitive decline is bidirectional: frailty accelerates cognitive deterioration, while MND itself contributes to the progression of frailty^13,14^. In the present study, more than half of the evaluated patients exhibited severe or very severe frailty, a finding consistent with the proportion of patients (55.05%) who had severe MND, suggesting a close relationship between frailty status and disease severity. Targeted interventions addressing frailty components may therefore help slow cognitive decline^13^ and preserve functionality and quality of life. Such interventions include reviewing polypharmacy, addressing falls^37^ and nutritional status, promoting deprescribing of inappropriate medications, ensuring adequate management of neurocognitive and neuropsychiatric symptoms, encouraging individualized multicomponent exercise programs, and facilitating the rehabilitation of sensory impairments, particularly hearing and vision^14^.

With regard to hospital stay, the most common primary diagnoses were infectious diseases, which are frequent in this population and have been associated in the literature with other geriatric syndromes, such as urinary incontinence and dysmobility syndrome^38^. The incidence of in-hospital delirium in our study (17.17%) was higher than that reported in previous studies, where the incidence among hospitalized patients with dementia is estimated at approximately 12%^39^. This finding may reflect the vulnerability of our sample, composed exclusively of nonagenarians with MND. In this very old population, severe frailty, multimorbidity, severe baseline functional impairment, and severe MND often coexist, creating a context in which acute medical illness and factors related to hospitalization can more easily precipitate delirium. However, given the descriptive design of the study, no causal inferences can be established.

These findings are consistent with recent international evidence in very old, hospitalized populations, including nonagenarians, demonstrating that patients aged ≥90 years experience markedly high rates of functional dependence, frailty, and delirium, particularly delirium superimposed on dementia, as well as increased in-hospital mortality compared with younger older adults. A recent systematic review and meta-analysis reported that delirium occurs in nearly half of hospitalized older adults with dementia and is associated with prolonged hospital stay, functional decline, and higher mortality, reinforcing the additive vulnerability conferred by advanced age and MND. Despite these similarities, data specifically focusing on hospitalized nonagenarians with MND in Latin America remain scarce, highlighting the importance of region-specific evidence to inform tailored geriatric care models for the rapidly expanding oldest-old population^12^.

Additionally, the length of hospital stay in our study was shorter than that described in other reports, where the average duration for patients with neurocognitive disorder ranges from 12^40^ to 17 days^41^. It is important to note that these referenced studies were conducted in the United Kingdom and Ireland, settings with distinct healthcare systems, hospital admission criteria, and demographic profiles compared to our Colombian context. Differences in discharge practices, availability of intermediate care facilities, social support structures, and health insurance models may all contribute to variations in length of stay across settings. In our institution, the early activation of home care plans, observed in 45.51% of patients, and the use of multidimensional geriatric assessment from admission may have facilitated earlier discharge in clinically stable patients. However, given the descriptive and cross-sectional design of this study, causal inferences cannot be drawn, and these observations should be interpreted with caution.

Finally, in the Latin American context, it is noteworthy that 45% of hospitalized patients had their chronic or palliative home-care plans activated or reactivated to ensure continuity of care within the health system. This remains a key area for improvement, as home-based care not only provides significant benefits for this population but also contributes to reducing healthcare costs, decreasing emergency admissions, and enhancing family support through better coordination with the healthcare system for both scheduled and urgent care^42^. Care for older adults remains fragmented and reflect ongoing challenges in care coordination in Colombia, especially during transitions between levels of care and the limited integration of interdisciplinary home-based management, as well as the need to strengthen geriatric education among physicians and other healthcare professionals.

This study has certain limitations. It is a descriptive cross-sectional study, and both the frequency and etiology of neurocognitive disorder were identified through self-report or proxy report by a family member, which may introduce reporting bias, although disease severity was clinically defined as previously described. Additionally, the study design limits the ability to establish causal relationships, and the sample, drawn from a single high-complexity hospital, may not be fully representative of the broader population with neurocognitive disorders. Furthermore, the absence of a cognitively normal comparison group limits the ability to contextualize the findings within the broader population of hospitalized nonagenarians. A comparative analysis between nonagenarians with and without MND represents a relevant direction for future research using the AGING registry data.

Nevertheless, a major strength of this study is the inclusion of a very old inpatient population with a detailed multidimensional geriatric assessment, which has rarely been described in Latin American contexts. The findings highlight the urgent need to strengthen comprehensive, interdisciplinary hospital care models for older adults with neurocognitive disorders in Latin America.

In conclusion, nonagenarians with MND in the studied population represent a vulnerable hospitalized population characterized by a high frequency of functional dependence, advanced frailty, a high prevalence of malnutrition, frequent geriatric syndromes such as delirium, and substantial in-hospital mortality. The predominance of severe cognitive impairment and infectious complications further underscores the clinical complexity observed in this group. These findings from a convenience sample at a single high-complexity center reinforce the need for comprehensive geriatric assessment, early identification of nutritional risk, and coordinated interdisciplinary care to optimize in-hospital management, discharge planning, and continuity of care for this particularly vulnerable population.

## Data Availability

The datasets generated and analyzed during the current study are available from the corresponding author upon reasonable request.
